# Method for obtaining reliable R-waves in fish electrocardiograms by utilizing conductivity of seawater

**DOI:** 10.1038/s41598-023-48262-7

**Published:** 2023-11-28

**Authors:** Natsuki Watanabe, Shinsuke Torisawa, Yasushi Mitsunaga, Masakazu Arima, Kazutaka Miyahara, Tsunemasa Saiki

**Affiliations:** 1 Graduate School of Agriculture, Kindai University, Nakamachi, 3327-204, Nara, 631-8505 Japan; 2 Graduate School of Engineering, Osaka Metropolitan University, 1-1, Gakuen-Cho, Naka-Ku, Sakai, 599-8531 Japan; 3 Fisheries Technology Institute, Hyogo Prefectural Technology Center for Agriculture, Forestry and Fisheries, 22-2, Minamifutami, Futami, Akashi, 674-0093 Japan; 4 Project Management Department, Hyogo Prefectural Institute of Technology, 3-1-12, Yukihira, Suma, Kobe, 654-0037 Japan; 5 Graduate School of Engineering, University of Hyogo, 2167, Shosha, Himeji, 671-2280 Japan

**Keywords:** Biological techniques, Ecology, Zoology, Ocean sciences, Health care, Engineering

## Abstract

A simple method for measuring bioelectric signals of fish in seawater is expected for managing the health of farmed fish and clarifying the ecophysiology of natural fish. We previously proposed a simple and unique method for measuring bioelectric signals of fish by inserting only one special internal electrode (which can be isolated from seawater) into the fish’s body and by sinking an external electrode in seawater (for utilizing the conductivity of seawater). However, the proposed method could not obtain fish electrocardiograms (ECGs) with reliable R-waves in the same manner as the conventional method. In this study, we thus experimentally investigated whether the R-waves of ECGs could be observed by optimizing the insertion position of the internal electrode into the fish’s body. The results of the experiment show that for four species of fish (each slightly longer than 10 cm) with different body shapes, reliable R-waves could be observed by inserting the internal electrode near the heart. We also investigated the possibility of simultaneously measuring ECGs of multiple fish by the proposed method. The results of the investigation show that the fish ECGs with R-waves of three fish could be observed simultaneously even when one single common external electrode replaced multiple external electrodes. This result indicates the advantage of the proposed method in reducing the total number of bioelectrodes compared to the conventional method for ECG measurements of multiple fish.

## Introduction

Owing to global warming, ocean pollution and overfishing, natural fish catches have been decreasing every year since the 1980s^1–4^. Under these circumstances, aquaculture will play a significant role in securing fishery resources^5–8^. To increase aquaculture production, not only the management of the breeding environment but also the health condition of the farmed fish are important factors.

The health condition of the farmed fish is mainly assessed by its length and weight^9,10^. Recently, the assessment of the health condition by cortisol and glucose noninvasively collected from the farmed fish is also explored^11–14^. In the assessment of rare fish species, there are the cases that a diver swims with a whale shark and measure fish cardiac activity by using an ultrasonic diagnostic apparatus^15,16^. Currently, a small implantable electronic device for aquatic animals (e.g., DST micro-HRT, Star-Oddi Ltd.) that can measure heart rate (BPM: beats per minute) based on an electrocardiogram (ECG) signal is available on the market^17–19^. In the near future, the health condition of fish may be assessed by non-real-time measurement using such an implantable device.

On the other hand, ECG measurements for fish have reported that an R-R wave interval becomes longer by receiving a sound stimulus^20^. Furthermore, in humans the predominant state of the sympathetic nerve and parasympathetic nerve has been assessed by frequency analysis based on R-R wave intervals^21–24^. The previously mentioned statements indicate the possibility of a new stress assessment for fish based on R-wave peak times of ECGs, and not only fish farmers but also marine biologists have expectations for this assessment.

**Figure 1:**
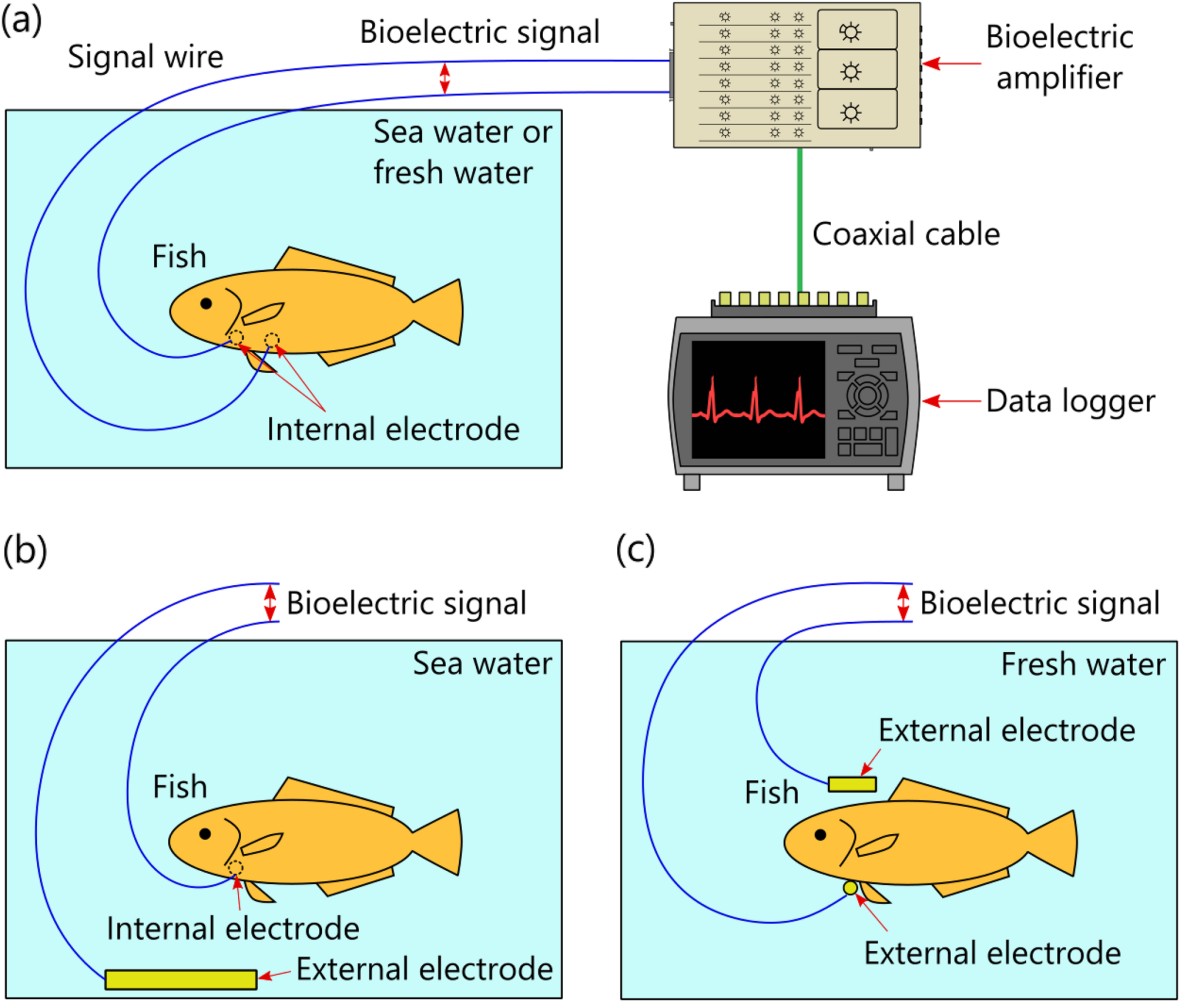
Principle of fish electrocardiogram (ECG) measurements in water: (**a**) conventional method using electrodes isolated from seawater or freshwater, (**b**) proposed method utilizing conductivity of seawater, and (**c**) other method utilizing freshwater without perfect insulator. The biological amplifier and data logger were drawn only in (**a**), and (**b**) and (**c**) were omitted.

In regard to conventional fish ECG measurement^25–30^ (Fig. 1a), to prevent electric short-circuiting between one pair of bioelectrodes via seawater (which is conductive), the bioelectrodes are embedded inside the living body of the fish under study by incision surgery, which can impose significant workload on the experimenters and physical stress on the fish. Considering those issues, we previously proposed a novel method^31^ for measuring bioelectric signals by utilizing the conductivity of seawater surrounding the entire body of the fish (Fig. 1b). As for the proposed method, an internal electrode is inserted into the fish’s body, an external electrode is sunk in seawater, and the bioelectric signal between the electrodes is measured. As the internal electrode, a special needle-type bioelectrode isolated from the seawater (by virtue of being inserted into the fish’s body) is used, invasive surgery is avoided. Therefore, when the ECGs of multiple fish are measured by using the proposed method, the usually required multiple external electrodes can be replaced by one common external electrode, thereby reducing the total number of bioelectrodes and, in turn, reducing the workload on the experimenters and the physical stress inflicted on the fish when a bioelectrode is inserted into the body of each fish. Incidentally, as for ECG measurement for freshwater fish, by taking advantage of the fact that freshwater is not a perfect insulator, it is theoretically possible to measure the bioelectric signal between a pair of bioelectrodes placed either side of the fish’s body (Fig. 1c). In a similar manner, human ECGs have been successfully measured by using a pair of bioelectrodes placed on the sides of a bathtub^32^. Although the proposed method for saltwater fish (Fig. 1b) has these advantages, unfortunately, we could not measure ECGs with the proposed method as clearly as those measured by the conventional method (Fig. 1a). However, in a previous study, we could observe the R-waves of human ECGs by using a method for non-invasive bioelectric measurement utilizing the conductivity of seawater^32^; accordingly, we thought that it would be highly likely that the proposed invasive method could observe the R-waves of fish ECGs, despite differences in skin structure.

In this study, we experimentally investigated whether clear ECGs with reliable R-waves could be measured with the proposed method by optimizing the insertion position of the internal electrode into the fish’s body. We also experimentally investigated whether clear ECGs for multiple fish could be measured simultaneously by measuring the bioelectric signal between the internal electrodes inserted at the optimized positions in the fish and the common external electrode.

## Relationship between inserting position of bioelectrode and measured ECG

### Experimental procedure

**Figure 2:**
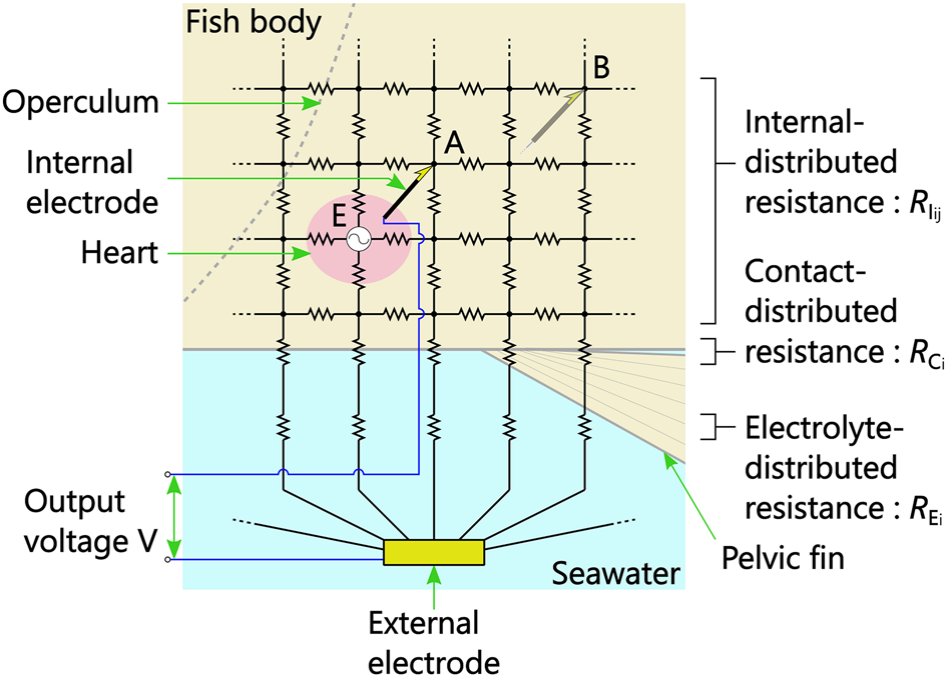
Electric-circuit model of bioelectric measurement utilizing conductivity of seawater. For ease of understanding, a simplified two-dimensional circuit is shown, although a three-dimensional circuit is used in reality.

Our previously proposed method for bioelectric measurement utilizing the conductivity of seawater simply measures the electric potential difference between an external electrode sunk in seawater and an internal electrode inserted into a fish’s body at two sites, namely above the gills and above the lateral line of the tail on the left hand side of the fish^31^. However, although the methodology is simple, as shown in Fig. 2, the measurement circuit is complex; in particular, an ECG measured from a bioelectric signal between internal and external electrodes is an action potential of the fish’s heart detected through multiple internal-, contact-, and electrolyte-distributed resistances. From this figure, it can be inferred that to induce the maximum action potential of the heart (to obtain maximum output voltage), the combined resistance between the internal and external electrodes should be small; in other words, the internal electrode should be inserted into the fish into near the heart (point A, not B in Fig. 2). Inducing a large action potential in this manner increases the possibility of observing R-waves of ECGs.

On the other hand, using the conventional method (namely, the bioelectrodes are isolated from seawater), Kojima et al.^27^ and Riyanto & Arimoto^30^ could observe reliable R-waves of ECGs by measuring the bioelectric signal between two needle-type bioelectrodes surgically inserted from the thoracoabdominal body surface of red seabream *Pagrus major* (Temminck and Schlegel, 1843) or jack mackerel *Trachurus japonicus* (Temminck and Schlegel, 1844)^27,30^. Considering this result, we thought that to observe the R-waves of ECGs by using the proposed method (utilizing the conductivity of seawater), the internal electrode should also be inserted from the thoracoabdominal body surface.

**Figure 3:**
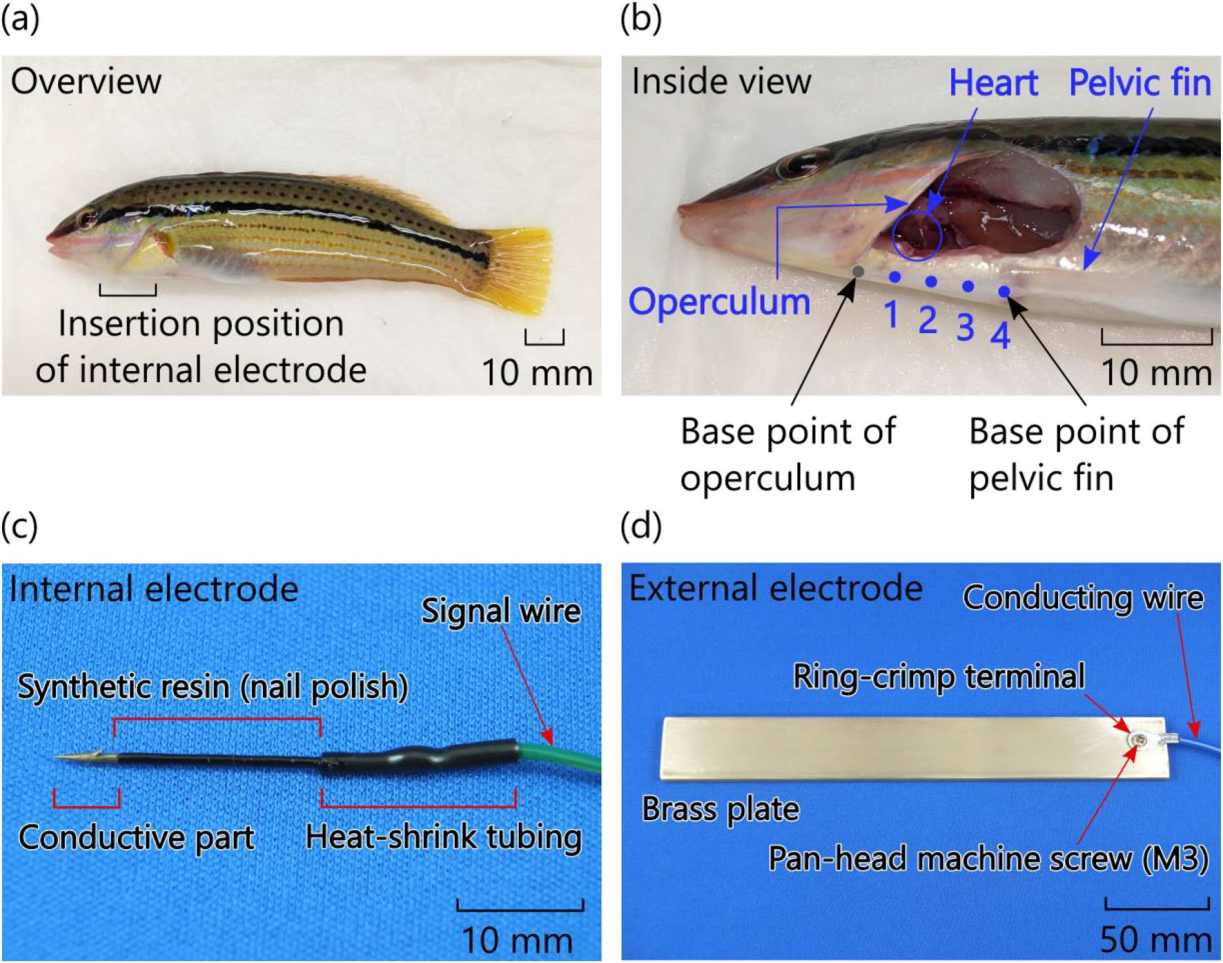
Photographs of multicolorfin rainbowfish (*Halichoeres poecilopterus*) subjected to practical bioelectric measurements and the bioelectrodes used in these measurements: (**a**) overview of the fish, (**b**) inside view of the fish, (**c**) fabricated internal electrodes and (**d**) fabricated external electrodes.

In this experiment, five multicolorfin rainbowfish *Halichoeres poecilopterus* (Richardson, 1846) (M1-M5) with a total length ranging from 11.0 to 16.5 cm (TL) and weight ranging from 14 to 59 g were subjected to practical bioelectric measurements (Fig. 3a). Note that all live fish were used in this study. This species was collected by rod and line from the shallow coastal waters of Awaji Island, Japan (34°23′21.5"N 134°53′43.5"E). Collection of this species was mostly carried out during the day as a result of their behaviour being that it spends most of the day sleeping buried in the sand, emerging occasionally in search of food. After collection, all individuals were put into a 30L fishing cooler filled with natural seawater supplied with air by pump, and were transported live to the laboratory, Hyogo Prefectural Institute of Technology. Then, the fish were held in two 57L tanks with sand in the bottom. Each tank was equipped with a system for filtering seawater and a protein skimmer. The laboratory had natural light and was constantly maintained at a temperature of 20 °C, suitable temperature for breeding conditions^33^, using air conditioning. Therefore, the seawater temperature in the tanks had been consistently maintained at 20 ± 0.2 °C. Additionally, once a week, one-third of the seawater in each tank was replaced with fresh seawater to prevent deterioration of water quality and to maintain a salinity concentration of 30 PPT. Before the experiment, the fish were raised for approximately one month. During this period, the fish were fed twice a week with a polychaete *Neanthes japonica* (Izuka, 1908) termed “gokai” in Japanese. No food was provided to the fish starting one day before the experiment.

As shown in Fig. 3b, we divided the distance from the base point of operculum to the base point of the pelvic fin of the fish into four, and then marked the division points “1,” “2,” and “3” and the base point of the pelvic fin “4” as the insertion points for the internal electrode. Note that the centre of the heart of the fish lies between insertion points 1 and 2. Incidentally, the distance between the two base points was about one-tenth of the total length of the fish.

The internal electrode (Fig. 3c) was made by connecting (soldering) a signal wire (AWG28, polyvinyl chloride wire, about 1 m long) to a nickel-and-gold-plated steel needle (30-mm long and 0.81-mm diameter) with a sharply pointed tip for inserting into the fish’s body. The connection was covered with a heat-shrink tube, and the electrode (except for the 5-mm-long tip) was coated with synthetic resin (commercial nail polish). In contrast, the external electrode (Fig. 3d) was a brass plate (with size of 170 × 25 × 5 mm^3^) connected to a conducting wire (AWG24) by a screwed ring-crimp terminal.

Immediately before the experiment, each individual was placed in an anesthetic solution, which was a 3000-fold dilution of the anesthetic (Sumitomo Pharma Animal Health Co., Ltd., FA100) with natural seawater, until the loss of equilibrium was observed. The loss of equilibrium was observed approximately 5 min after immersion. After lifting the fish from the anesthetic solution, the internal electrode was inserted into the thoracoabdominal region of the fish’s body to a depth of 7 mm at each insertion point. The depth for inserting the internal electrode at 7mm was determined by dissecting three individuals and then confirming the position of the heart. The fish was then put into a plastic tub (with opening diameter of about 36 cm) filled with 3 L of conductive solution (water temperature: 20 °C; electrical conductivity: 4.6 S/m) in which the external electrode was sunk. To sustain the effect of anaesthesia, a conductive solution containing a 9000-fold dilution of the anaesthetic was used. The internal electrode was thus prevented from falling off the fish’s body by the restriction of movement to the fish. In the next ten minutes, the internal electrode was sequentially inserted into each measurement point of the fish and each ECG was measured for 1 min. After the measurements were completed, the fish were immediately euthanized. During the experiment, the conductive solution in the plastic tub was continuously supplied with air by pump, and its temperature was indirectly controlled by an air conditioner. Experiment was conducted during daylight hours under fluorescent lighting (200–300 lx).

With the entire body of the fish surrounded by the conductive solution, the bioelectric signal between the internal electrode inserted at each insertion point and the external electrode was measured. Incidentally, resistances between the internal electrode and the external electrode and between the internal electrode left in seawater and the external electrode were about 0.8 and 0.2 kΩ, respectively. These resistances were measured by a resistance meter specialized for bioelectric measurement (Nihonsanteku Co., MaP811). The bioelectric signal detected between the electrodes was amplified a thousand times and processed with high- and low-pass filters (passing frequency: 5 to 30 Hz) by a bioelectric amplifier (TEAC Co., BA1008) (Fig. 1a). The amplified and processed bioelectric signals were then recorded by a data logger (Graphtec Co., GL900) (Fig. 1a) for one minute.

This study was specifically approved by Kindai University Committee on Animal Research and Bioethics (Permit Number: KAAG-2021–002) and was carried out in strict accordance with the Guiding Principles for the Care and Use of Research Animals, including ARRIVE guidelines^34^. The laboratory fish were also kept under the FELASA EUROGUIDE^35^ and treated according to the AVMA guideline (2020 Edition)^36^. Incidentally, according to Japanese law (Act on Welfare and Management of Animals), fish are not included in the list of experimental animals; thus, unfortunately there are no relevant institutional and national guidelines for the care and use of laboratory fish.

### Results and discussion

**Figure 4:**
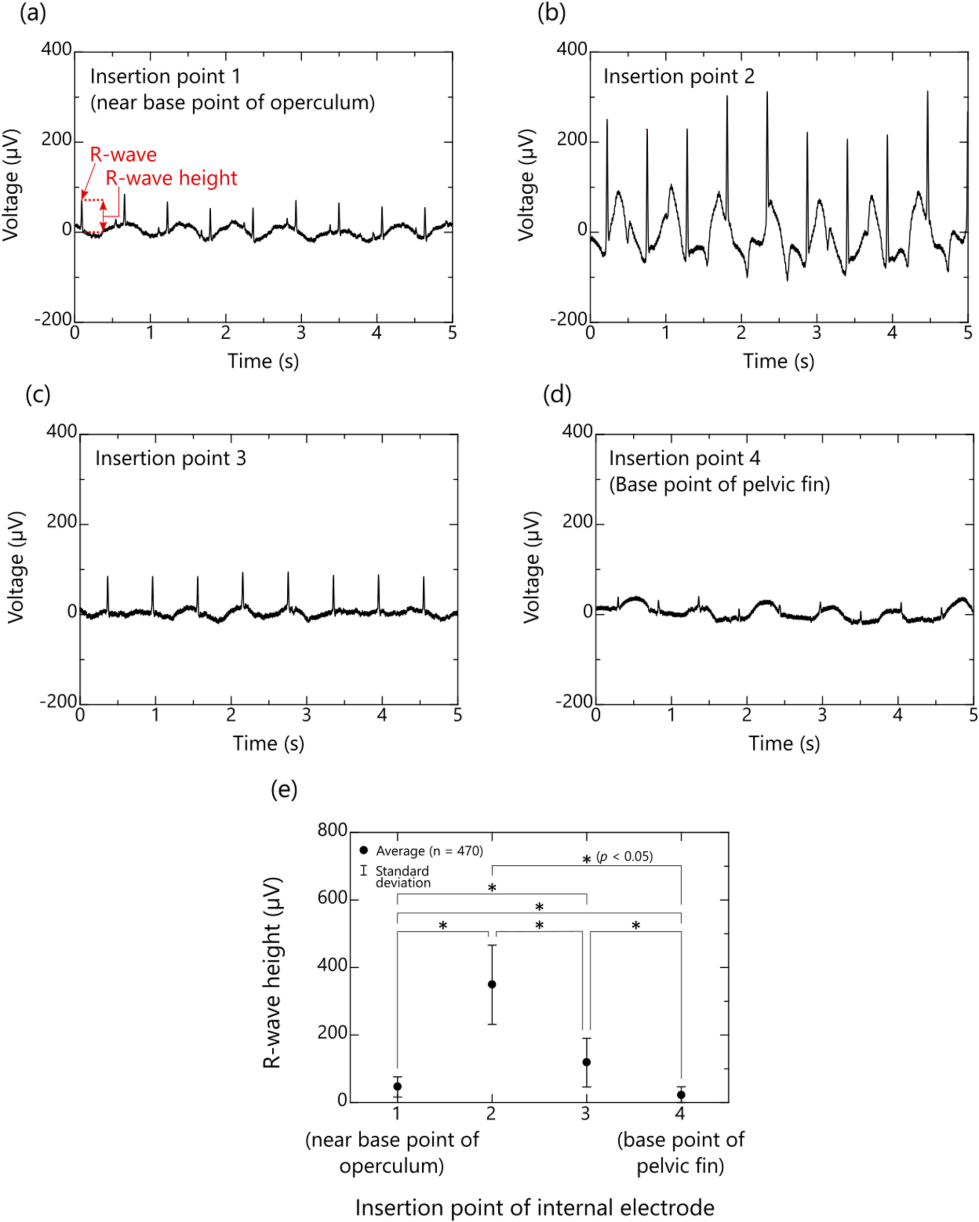
Measured bioelectric signals of multicolorfin rainbowfish between an internal electrode inserted from the thoracoabdominal body surface and an external electrode sunk in seawater: (**a**)–(**d**) typical waveforms of bioelectric signals detected by internal electrodes inserted from points 1 (near base of operculum) to 4 (base of pelvic fin) of the same fish (subject: M1) and (**e**) average and standard deviation of R-wave heights of ECGs obtained from five fish (subject: M1–M5) at each insertion point (Modified Sequentially Rejective Bonferroni Procedure, *p < 0.05, *n* = 5 individuals × 94 of R-wave heights (total: 470)).

Typical examples of the bioelectric signals measured at thoracoabdominal insertion points 1 to 4 of the same fish (multicolorfin rainbowfish, M1) are shown in Fig. 4a–d, respectively. These figures show that, even if the fish is in low-activity condition under anesthesia, the R-waves of the ECG can be observed at all the insertion points of the internal electrode. They also show that the R-wave heights increase from insertion point 1 to 2 (Fig. 4a and b) and then gradually decrease from insertion point 2 to 4 (Fig. 4c and d). Note that R-wave height is defined as the seawater electric potential (0 V) (as a reference) subtracted from the electric potential of the peak R-wave (which indicates ventricular myocardial activity).

The average and standard deviation of the R-wave heights of the five fish for each insertion position of the internal electrode are summarized in Fig. 4e. Here, the average and standard deviation were calculated from about 100 R-wave height data included in one minute ECG of each fish. Average R-wave height obtained at insertion points 1 to 4 were respectively 24, 298, 76, and 13 µV and their standard deviations were respectively 51, 109, 27, and 12 µV. Furthermore, statistical analysis was conducted using one-way repeated measures ANOVA adjusted by Greenhouse–Geisser's Epsilon and Shaffer's Modified Sequentially Rejective Bonferroni Procedure to compare the significance between each insertion point, revealing significant differences in all combinations. These results indicate that internal-electrode insertion point 2, where the R-wave heights of the fish were large, albeit varied widely, is the most suitable for measuring ECGs by the proposed method utilizing the conductivity of seawater.

As can be seen from the inside view of one of the fish (Fig. 3b), the needle tip (conductive part) of the internal electrode inserted from point 2 corresponded to the electrode position capable of obtaining the maximum action potential of the heart inferred from the theoretical circuit model (Fig. 2). Note that the resistance distribution of each part of the fish is different, so even if the distance from the heart is the same, the R-wave height is not necessarily the same.

## Measured ECGs of fish species with different body shapes

### Experimental procedure

According to the experimental results presented in the previous section, for a certain fish species (multicolorfin rainbowfish with spindle body shape), to observe reliable R-waves of ECGs by the proposed method (using the conductivity of seawater), the conductive part (tip) of the internal electrode must be placed near the heart. Therefore, to confirm this fact, we investigated fish species with different body shapes from the species used in the experiment with the aim of determining whether reliable R-waves could be observed in the same way.

In this investigation, three fish of three species (*n* = 9) distributed near the coast of Japan were subjected to ECG measurement: Japanese black rockfish *Sebastes cheni* (Barsukov, 1988) [J1 – J3; TL = 9.0–9.5 cm; weight = 18–23 g] with laterally-flattened body shape; flounder *Paralichthys olivaceus* (Temminck & Schlegel, 1846) [F1 – F3; TL = 13.0–14.0 cm; weight = 27–36 g] with flattened body shape and devil stinger *Inimicus japonicus* (Cuvier, 1829) [D1 – D3; TL = 12.0–14.5 cm; weight = 53–104 g] with longitudinally flattened body shape. All the test fish were artificially produced and reared in culture in Hyogo Prefectural Technology Center for Agriculture, Forestry and Fisheries. The ECGs of these fish species were measured in basically the same way described in the previous section, using the proposed method; however, the insertion point and depth of the internal electrode differed for each fish species because the internal electrodes must be inserted near the ventricles. Specifically, the insertion point in the Japanese black rockfish was two-thirds from the base point of the operculum to that of the pelvic fin, and its insertion depth was 11 mm; that in the flounder was at the base point of the pelvic fin, and its insertion depth was 16 mm; and that of the devil stinger was four-fifths from the base point of the operculum to that of the pelvic fin, and insertion depth was 15 mm. The location of the ventricles of the heart in these fish species was confirmed by autopsy, and the resistance between the internal and external electrodes varied from 0.5 to 1.2 kΩ, depending on the species and individual fish.

### Results and discussion

**Figure 5:**
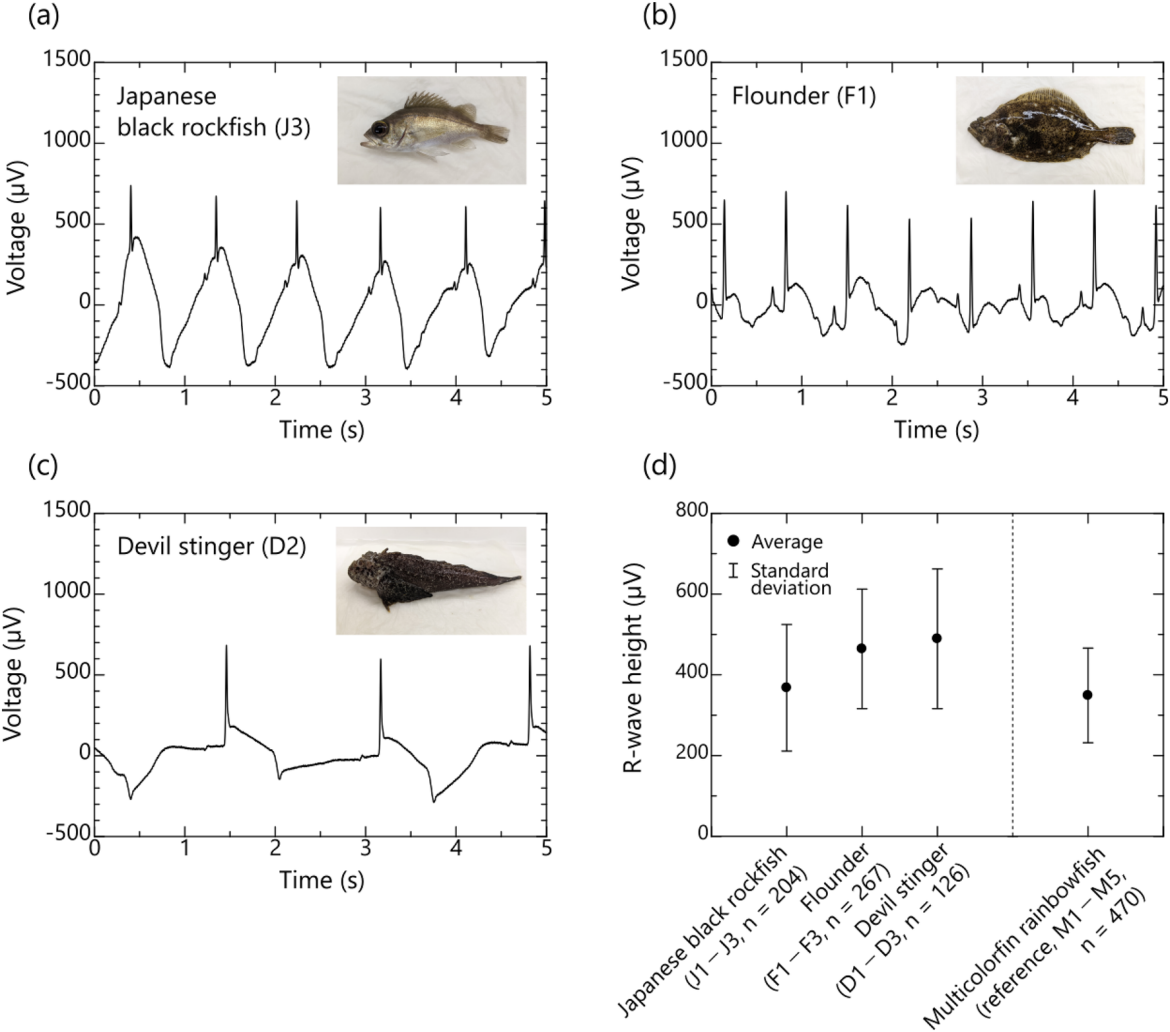
Bioelectric signals of three fish species obtained by the proposed method utilizing conductivity of seawater: (**a**)–(**c**) typical waveforms of bioelectric signals of Japanese black rockfish (*Sebastes chen*i, J3) with laterally flattened body shape, flounder (*Paralichthys olivaceus,* F1) with flattened body shape, and devil stinger (*Inimicus japonicus,* D2) with longitudinally flattened body shape (For ease of recognition of the body shape of each fish species, a photograph is inserted in the upper right of each figure.), and (**d**) average and standard deviation of the R-wave height of each fish species (Japanese black rockfish: n = 3 individuals × 68 of R-wave heights (total: 204), flounder: n = 3 individuals × 89 of R-wave heights (total: 267), devil stinger: n = 3 individuals × 42 of R-wave heights (total: 126)).

Typical examples of bioelectric signals of Japanese black rockfish, flounder, and devil stinger obtained by the proposed method are shown in Fig. 5a–c, respectively. The measurement time was 1 min in this experiment, which was the same duration during which approximately 100 beats of data were obtained at each insertion position of each individual in multicolorfin rainbowfish. Average and standard deviation of R-wave height for 3 fish are summarized for each fish species in Fig. 5d. Average R-wave heights for Japanese black rockfish, flounder, and devil stinger were 366, 464, and 488 µV, respectively, and their standard deviations were 157, 148, and 177 µV, respectively. These values indicate that the same or clearer ECGs can be obtained for Japanese black rockfish, flounder, and devil stinger when the insertion point is situated close to the heart as seen by insertion point 2 for multicolorfin rainbowfish. This shows that the proposed method (utilizing the conductivity of seawater) can obtain reliable R-waves of ECGs for various saltwater-fish species.

## Simultaneous ECG measurements for multiple fish

**Figure 6:**
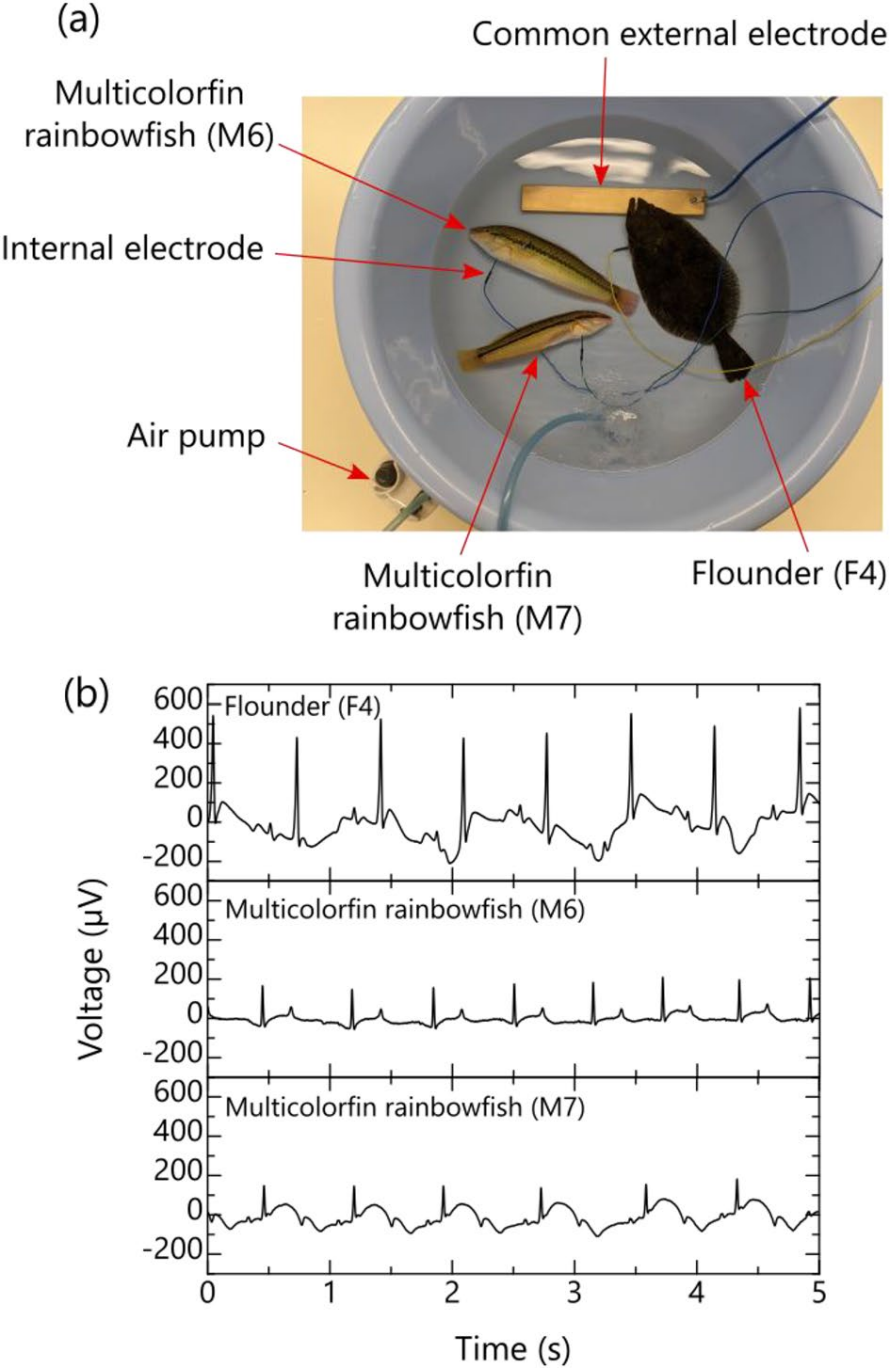
Simultaneous ECG measurement of multiple fish by the proposed method utilizing conductivity of seawater: (**a**) photograph of multiple fish during the experiment and (**b**) typical waveforms of bioelectric signals of multiple fish.

According to the experimental results described in the previous section, the proposed method (using the conductivity of seawater) can obtain clear ECGs for various fish species in a similar manner to the conventional method. Therefore, we experimentally attempted to measure, simultaneously, multiple fish ECGs of different species by taking advantage of a key feature of the proposed method, namely, the ability to replace multiple external electrodes with one common external electrode. In this experiment, two multicolorfin rainbowfish (M6 = 15.5 cm TL and M7 = 14.5 cm TL) and one flounder (17.5 cm TL) F4 were subjected to ECG measurements. After the internal electrode was inserted near the ventricle of the heart of each fish, the bioelectric signal between the internal electrode of each fish and the common external electrode was measured as shown in Fig. 6a. If experimental conditions can be properly established (e.g., ensuring that the fish remains still and the insertion electrode position remains stable), there is a possibility of obtaining a reliable R-waveform similar to Fig. 6b. Incidentally, as for the flounder (see Fig. 6b), the R-wave heights of 464 µV were about the same as the average shown in Fig. 5d. For the multicolorfin rainbowfish, however, they were about half of the average (149 µV) at the insertion point 2 in Fig. 4e. The reason for this difference in heights is that the insertion point of the internal electrode in the multicolorfin rainbowfish was slightly shifted from point 2 to point 3, and even this slight shift may have affected the distributed resistance, resulting in a difference in the R-wave heights.

Regardless of single or multiple fish under ECG measurement, the proposed method (utilizing the conductivity of seawater) is a human-friendly (workload less) and fish-friendly (less stressful) bioelectric measurement that requires fewer bioelectrodes inserted into the fish. Therefore, in investigations such as evaluation of fish stress based on R-R intervals of ECGs, the proposed method can provide a better measurement environment than the conventional method with less experimental problems such as entangled signal lines and less stress on the fish caused by insertion of bioelectrodes.

## Conclusion and future work

We previously proposed a simple and unique method for measuring fish bioelectric signals by inserting only one special internal electrode (which can be isolated from seawater) into the fish’s body and by sinking an external electrode in seawater (for utilizing the conductivity of seawater). In this study, to solve the pending issue concerning the proposed method at that time, we experimentally investigated whether reliable R-waves of fish ECGs could be observed in a similar manner to the conventional method by optimizing the insertion position of the internal electrode into the fish’s body. The results of the experiment revealed that for four species of fish (each slightly longer than 10 cm) with different body shapes, reliable R-waves could be observed by inserting an internal electrode near the ventricle of the heart. Next, we investigated the possibility of simultaneous ECG measurement by the proposed method with multiple fish. The results of the investigation show that the R-waves of each of three fish could be observed, even when one single common external electrode replaced the multiple external electrodes. This result indicates the advantage of the proposed method in reducing the total number of bioelectrodes compared to the conventional method for ECG measurements on multiple fish.

In the future, after devising a technique for fixing the position of the internal electrode even if the fish moves, we plan to use the proposed method to investigate the R-R interval of ECGs of multiple fish of different species and genders. In addition, considering the knowledge of physical and mental stress on individual fish obtained from this investigation, we hope to clarify the relationship between such fish stress and fish behavior.

### Supplementary Information

Supplementary Information.

## Data Availability

The data that support the findings of this study are available from the corresponding author upon reasonable request.
