# Supplementary material for: Method for obtaining reliable R-waves in fish electrocardiograms by utilizing conductivity of seawater
Source: Sci Rep. 2023 Nov 28;13:20994. doi: 10.1038/s41598-023-48262-7 (PMC10684608; doi:10.1038/s41598-023-48262-7)
Supplement: Supplementary file 1 — Supplementary Information. [file 41598_2023_48262_MOESM1_ESM.pdf]

Supplementary materials

1) ECGs of multicolorfin rainbowfish obtained from each point of each individual for entire sampling period: (a) M1, (b) M2, (c) M3, (d) M4 and (e) M5. Note that these ECGs were not measured simultaneously.

(a)

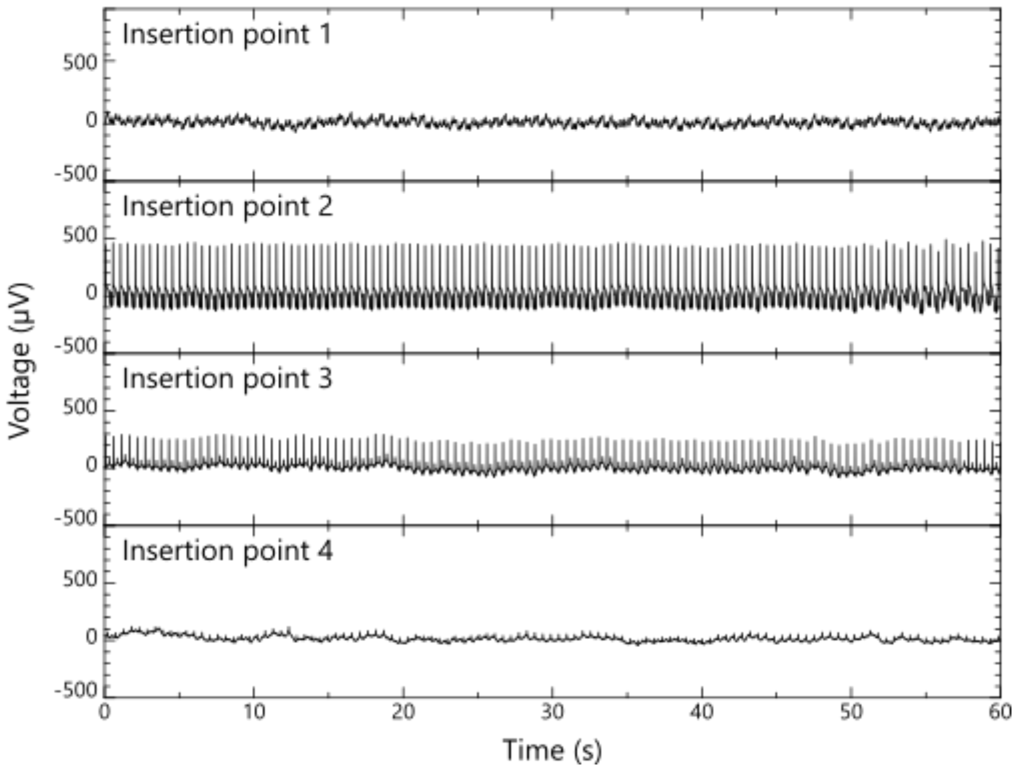

(b)

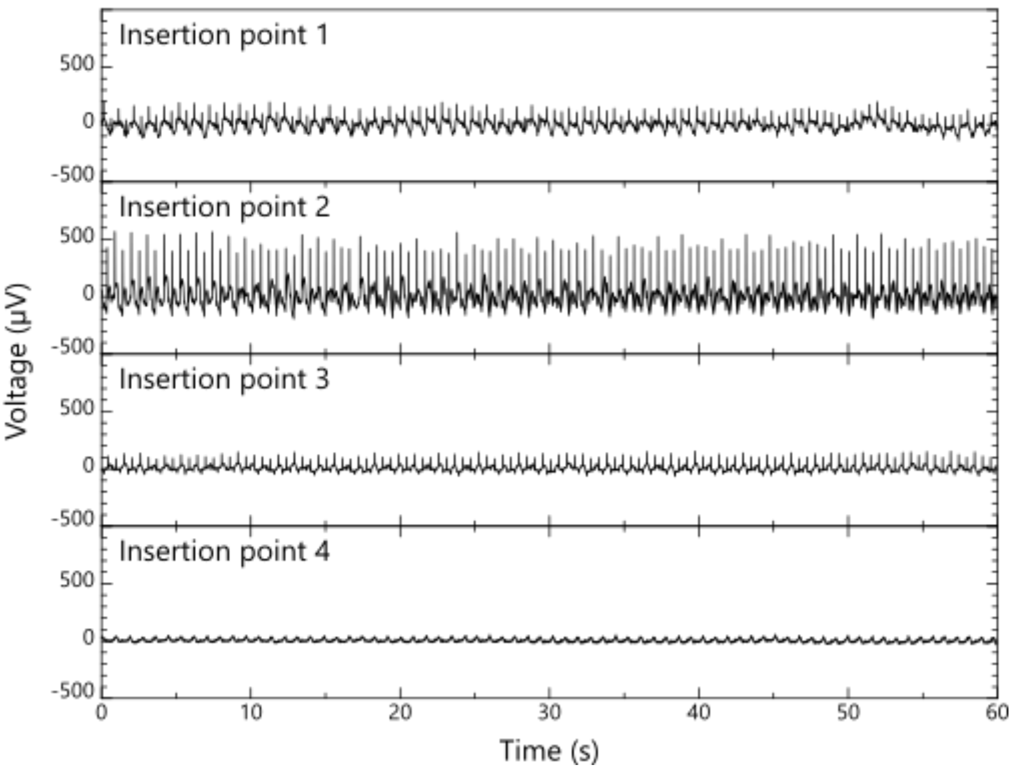
Insertion point 1
Insertion point 2
Insertion point 3
Insertion point 4
500
0
-500
Voltage (µV)
0
10
20
30
40
50
60
Time (s)

(c)

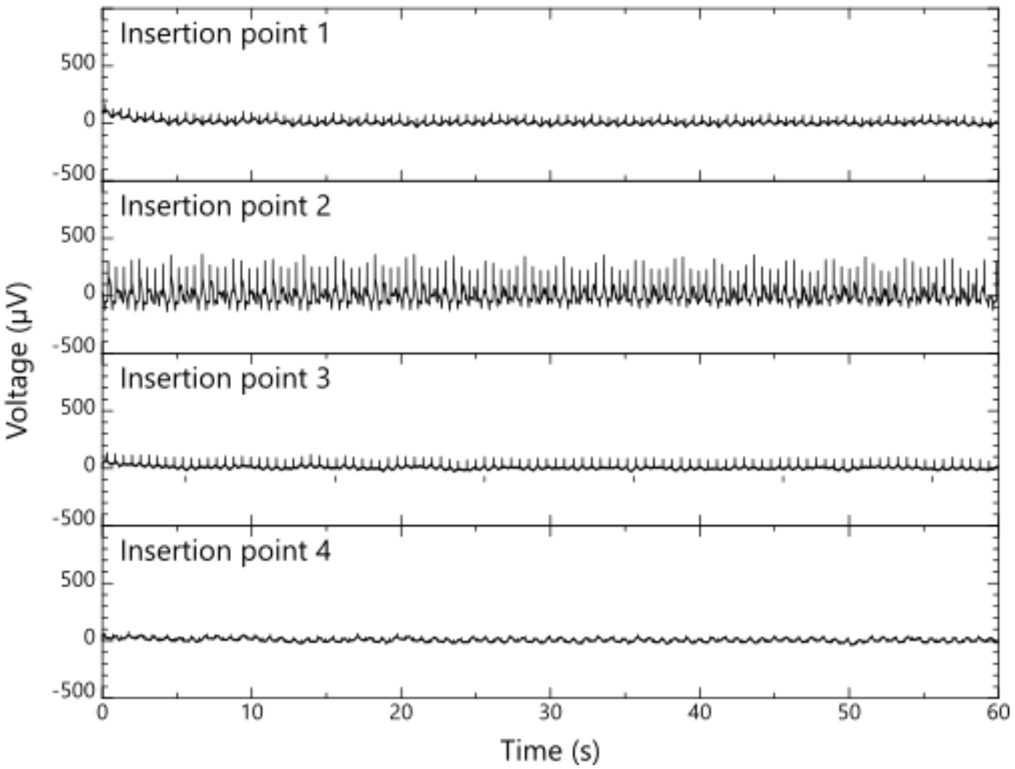
Insertion point 1
Insertion point 2
Insertion point 3
Insertion point 4
500
0
-500
Voltage (μV)
0
10
20
30
40
50
60
Time (s)

(d)

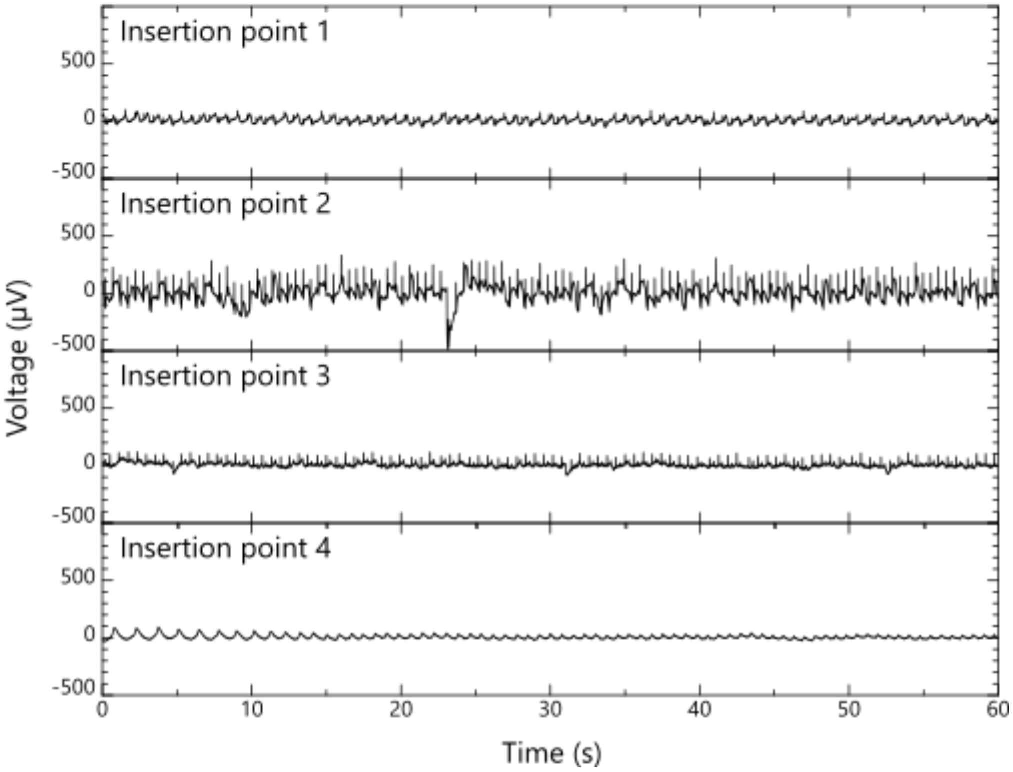
Insertion point 1
Insertion point 2
Insertion point 3
Insertion point 4
500
0
-500
Voltage (μV)
0
10
20
30
40
50
60
Time (s)

(e)

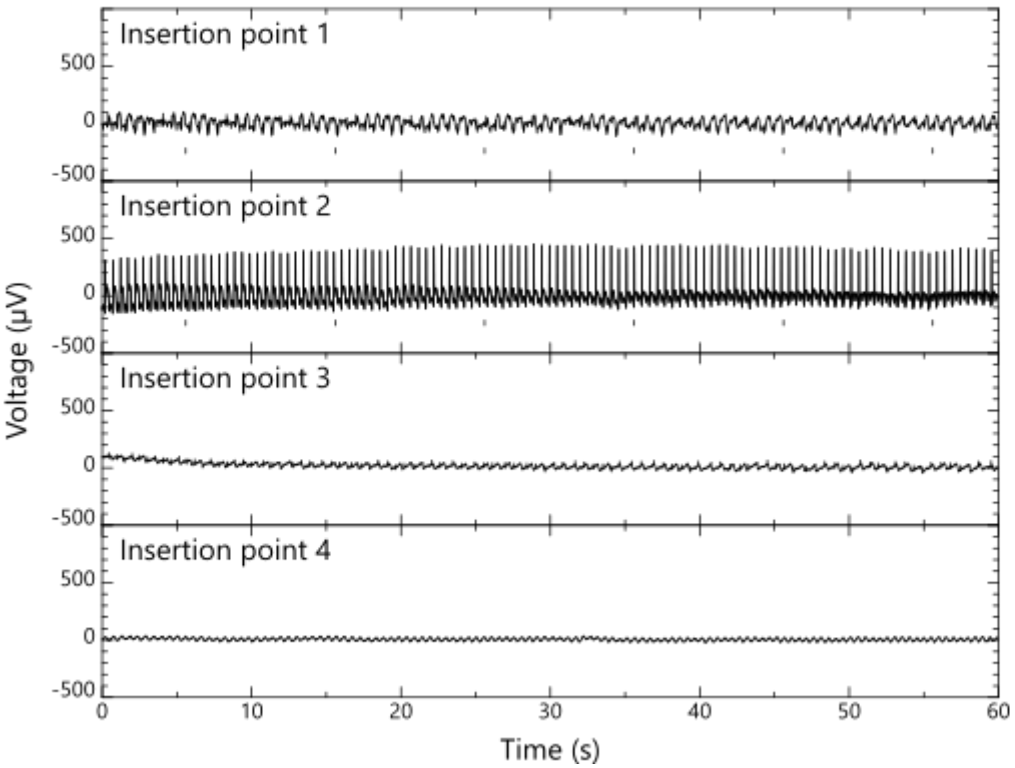
Insertion point 1
Insertion point 2
Insertion point 3
Insertion point 4
Voltage (μV)
500
0
-500
0
10
20
30
40
50
60
Time (s)

2) Statistical analyses based on ECGs of multicolorfin rainbowfish obtained from each point.

(a)

<< DESCRIPTIVE STATISTICS >>

Groups (Insertion points)	n (Individuals x R wave heights)	Mean	S.D.
1	470 (5 x 94)	46.3	29.9
2	470	348.8	117.3
3	470	118.2	72.1
4	470	21.5	25.2

(b)

<< SPHERICITY INDICES >>

== Mauchly's Sphericity Test and Epsilons ==

Effect	W	approx.Chi	df	p	LB	GG	HF	CM
Insertion points	0.1262	968.0957	5	< 0.01	0.3333	0.551	0.5527	0.5527

LB = lower.bound, GG = Greenhouse-Geisser, HF = Huynh-Feldt-Lecoutre, CM = Chi-Muller

(c)

<< one-way Repeated Measures ANOVA TABLE >>

== Adjusted by Greenhouse-Geisser's Epsilon ==

Source	Sum Sq	df	Mean Sq	F-ratio	p-value
Within Groups	3,506,060.4	469.0	7,475.6		
Between Groups	31,375,013.1	1.7	18,979,137.1	2413.8	< 0.01
Residuals	6,096,150.1	775.3	7,862.8		
Total	40,977,223.6	1,879.0	21,808.0		

(d)

<< POST ANALYSES >>

== Shaffer's Modified Sequentially Rejective Bonferroni Procedure ==

Pair	Diff	t-value	df	p	adj.p		
2-4	327.4	63.9	469	< 0.05	< 0.05	2 > 4	*
1-2	- 302.5	52.0	469	< 0.05	< 0.05	1 < 2	*
2-3	230.6	43.5	469	< 0.05	< 0.05	2 > 3	*
3-4	96.8	36.5	469	< 0.05	< 0.05	3 > 4	*
1-3	- 71.9	20.2	469	< 0.05	< 0.05	1 < 3	*
1-4	24.9	15.2	469	< 0.05	< 0.05	1 > 4	*

Alpha level is 0.05.
